# TZD-Based Hybrid Molecules Act as Dual Anti-*Mycobacterium tuberculosis* and Anti-*Toxoplasma gondii* Agents

**DOI:** 10.3390/ijms24032069

**Published:** 2023-01-20

**Authors:** Katarzyna Dzitko, Barbara Kaproń, Agata Paneth, Adrian Bekier, Tomasz Plech, Piotr Paneth, Nazar Trotsko

**Affiliations:** 1 Department of Molecular Microbiology, Faculty of Biology and Environmental Protection, University of Lodz, 90-237 Lodz, Poland; 2 Department of Clinical Genetics, Medical University of Lublin, 20-080 Lublin, Poland; 3 Department of Organic Chemistry, Medical University of Lublin, 20-093 Lublin, Poland; 4 Department of Pharmacology, Medical University of Lublin, 20-080 Lublin, Poland; 5 Institute of Applied Radiation Chemistry, Faculty of Chemistry, Lodz University of Technology, 90-924 Lodz, Poland

**Keywords:** thiazolidinedione, thiosemicarbazone, pyridine-4-carbohydrazone, dual anti-*Mycobacterium tuberculosis* and anti-*Toxoplasma gondii* mode of action, in vitro and in vivo toxicity, PAMPA-BBB assay

## Abstract

Two distinct intracellular pathogens, namely *Mycobacterium tuberculosis (Mtb)* and *Toxoplasma gondii (Tg),* cause major public health problems worldwide. In addition, serious and challenging health problems of co-infections of *Tg* with *Mtb* have been recorded, especially in developing countries. Due to this fact, as well as the frequent cases of resistance to the current drugs, novel anti-infectious therapeutics, especially those with dual (anti-*Tg* and anti-*Mtb*) modes of action, are needed. To address this issue, we explored the anti-*Tg* potential of thiazolidinedione-based (TZD-based) hybrid molecules with proven anti-*Mtb* potency. Several TZD hybrids with pyridine-4-carbohydrazone (PCH) or thiosemicarbazone (TSC) structural scaffolds were more effective and more selective than sulfadiazine (SDZ) and trimethoprim (TRI). Furthermore, all of these molecules were more selective than pyrimethamine (PYR). Further studies for the most potent TZD-TSC hybrids **7**, **8** and **10** and TZD-PCH hybrid molecule **2** proved that these compounds are non-cytotoxic, non-genotoxic and non-hemolytic. Moreover, they could cross the blood–*brain barrier* (BBB), which is a critical factor linked with ideal anti-*Tg* drug development. Finally, since a possible link between *Tg* infection and the risk of glioblastoma has recently been reported, the cytotoxic potential of TZD hybrids against human glioblastoma cells was also evaluated. TZD-PCH hybrid molecule **2** was found to be the most effective, with an IC_50_ of 19.36 ± 1.13 µg/mL against T98G cells.

## 1. Introduction

Two distinct intracellular pathogens, namely *Mycobacterium tuberculosis (Mtb)* and *Toxoplasma gondii (Tg),* cause major public health problems worldwide; *Mtb* is the human etiologic agent of tuberculosis (TB), while the zootonic parasite *Tg* causes serious health problems in immunocompromised people and developing fetuses. Both infections share many characteristics; they can cause acute disease, or they can be latent and asymptomatic. Latent infections represent the majority of the cases after infection with either of these two pathogens. Latency could be explained by the capacity of *Mtb* and *Tg* pathogens to survive or even replicate within macrophages or other target cells, and their capacity to shield themselves from the immune response by residing within a non-fusogenic vacuole inside the macrophage. However, in immunocompromised people, both infections manifest as acute disease [1]. As per the estimation of the World Health Organization, *Mtb* infection is the thirteenth leading cause of death and the second leading cause of mortality from infectious diseases after COVID-19; in 2020, a total of 10 million people fell ill with TB and 1.5 million people died from this infectious disease [2]. Incorrect medication prescription by health care providers, patient non-compliance with drug regimens, and poor-quality drugs have led to the emergence of resistant *Mtb* strains, ranging from multidrug- [3,4] to fully drug-resistant TB [5,6]. These aspects coupled with toxicities, numerous side effects, and long-term treatment regiments associated with the use of current TB medicines indicate that new drugs are urgently needed [7,8,9,10,11,12]. In a similar manner to *Mtb*, the *Tg* pathogen infects one third of the human population and has co-evolved with the human population for centuries [13,14]. Although *Tg* infection in immunocompetent individuals is usually asymptomatic or minor and self-*limited* [15,16,17], in patients with profound T-cell deficiency, it can cause serious complications, or even be fatal [18,19,20]. Furthermore, congenital toxoplasmosis, which occurs during pregnancy, can cause spontaneous abortions, stillbirths, and some degrees of mental or physical retardation, hydrocephalus, microcephalus, intracerebral calcifications, blindness, and deafness [21]. The current gold-standard treatments, based on a combination of pyrimethamine and sulfonamide, are associated with adverse side effects, mostly pyrimethamine-related bone marrow suppression, intolerance or allergic reactions to the sulfonamide component [22,23,24,25,26,27]. Alternative therapies, such as macrolides, atovaquone, and cotrimoxazole, also have several side effects [28,29,30]. Furthermore, serious and challenging health problems of co-infections of *Tg* with *Mtb* have been recorded in some cases, especially in developing countries. These include ocular co-infection [31], cerebritis from intracranial toxoplasmosis and tuberculosis [32], cerebral toxoplasmosis combined with disseminated tuberculosis [33], neurotoxoplasmosis mimicking intracranial tuberculoma [34,35], pulmonary tuberculosis co-infection with toxoplasmosis [36], and others [37,38,39,40,41]. In particular, patients with previously diagnosed TB are at a high risk for the acquisition of *Toxoplasma* infection, which could reactivate the latent toxoplasmosis [42]. Therefore, alternative therapies with dual anti-infectious modes of action are needed to overcome this problem.

**Figure 1:**
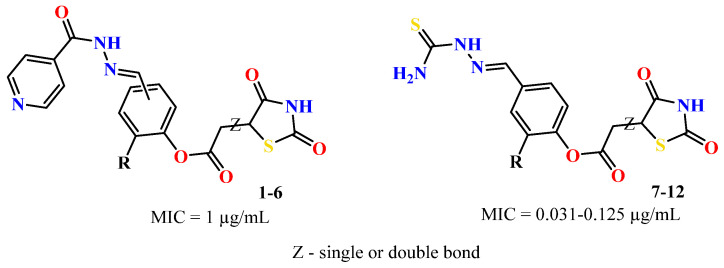
TZD-PCH (**1**–**6**) and TZD-TSC (**7**–**12**) hybrid molecules with promising anti-*Mtb* potency [43,44,45]. For the numbers used to identify the hybrid molecules (**1**–**12**), see Table 1.

To address this issue, we explored the anti-*Mtb* modes of action of promising thiazolidinedione (TZD) molecular hybrids with negligible cytotoxic effects against human cells, expressed by a rigorous 30% cytotoxic concentration (CC_30_) parameter. Several TZD-based hybrids that incorporate pyridine-4-carbohydrazone (PCH) [43] or thiosemicarbazone (TSC) [44,45] and structural fragments have been recently reported and evaluated for their anti-*Mtb* activity. Some of these molecules (**1**–**12**) revealed excellent in vitro potency with minimal inhibitory concentrations (MICs) in the range of 0.031–1 µg/mL (Figure 1). Moreover, the most potent hybrid molecules were more effective and more selective for the *Mtb* pathogen than the reference drug rifampicin, indicating their potential as anti-mycobacterial agents. To extend these preliminary findings with details on the possible anti-*Tg* effect of TZD-based hybrid molecules, in vitro anti-*Toxoplasma* studies were subsequently undertaken and their promising results are presented in this paper. Several compounds were found to be more effective and more selective than sulfadiazine, trimethoprim, and pyrimethamine. Further studies for the most potent of them proved that they are non-cytotoxic, non-genotoxic and non-hemolytic. Moreover, they could cross the BBB, which is a critical factor linked with ideal anti-*Tg* drug development. Finally, since the possible link between *Tg* infection and the risk of glioblastoma has recently been reported [46], the cytotoxic potential of selected TZD hybrids against human glioblastoma cells was also evaluated. Among them, TZD-PCH hybrid molecule **2** was found to be the most effective, with an IC_50_ of 19.36 ± 1.13 µg/mL against T98G cells. As far as we are aware, the dual anti-infectious (anti-*Tg* and anti-*Mtb*) properties for TZD-based hybrid molecules have not been recorded. 

## 2. Results and Discussion

### 2.1. Chemistry

**Figure 2:**
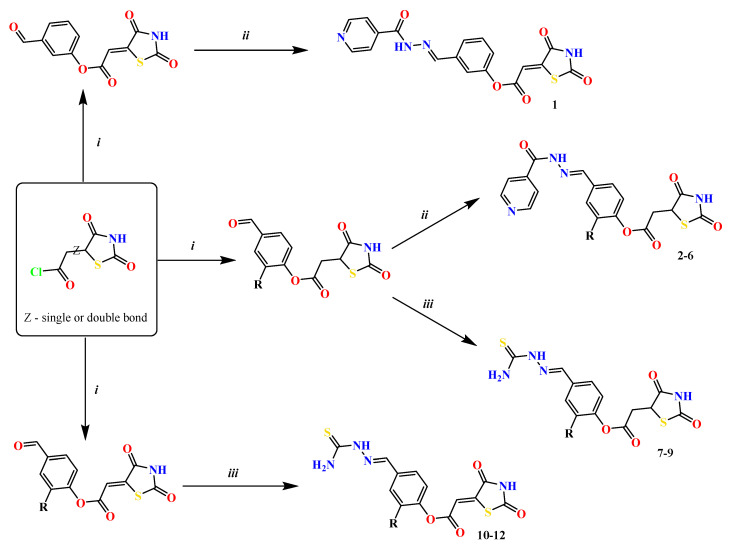
Synthetic route for TZD-PCH (**1**–**6**) and TZD-TSC (**7**–**12**) hybrid molecules. Reagent and conditions*:* (*i*) corresponding hydroxybenzaldehyde, pyridine, 1,4-dioxane, rt, 2h, acidified with dilute HCl; (*ii*) isonicotinic acid hydrazide, anhydrous ethanol, reflux; (*iii*) TSC, anhydrous ethanol, reflux. For the numbers used to identify the TZD hybrid molecules (**1**–**12**), see Table 1.

The previously reported TZD-PCH (**1**–**6**) and TZD-TSC (**7**–**12**) hybrid molecules were resynthesized according to a two-step synthetic procedure described in detail in Refs. [43,44,45], respectively. Briefly, a condensation reaction of (2,4-dioxo-1,3-thiazolidin-5-yl/ylidene)acetyl chlorides with hydroxybenzaldehydes afforded formylphenyl (2,4-dioxo-1,3-thiazolidin-5-yl/ylidene)acetates. Further reactions with isonicotinic acid hydrazide or thiosemicarbazide produced the final TZD-PCH (**1**–**6**) and TZD-TSC (**7**–**12**) hybrid molecules (Figure 2).

### 2.2. Inhibition of T. gondii Tachyzoites In Vitro

**Figure 3:**
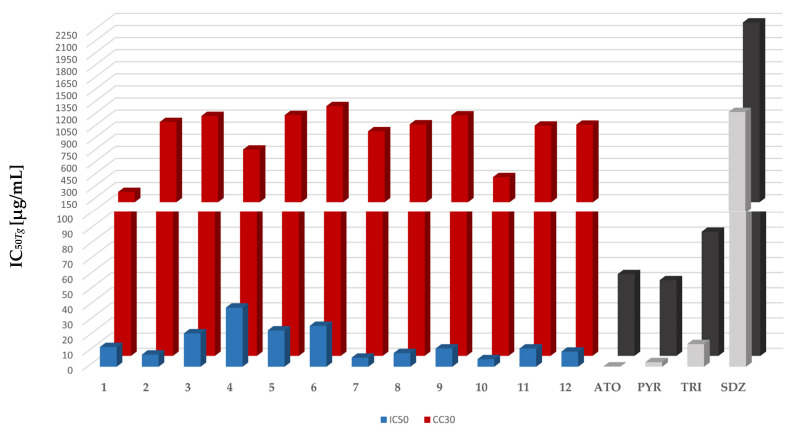
Anti-*Tg* activity (IC_50*Tg*_), cytotoxicity (CC_30_), and selectivity index (SI) of TZD hybrid molecules (**1**–**12**). ATO—atovaquone, PYR—pyrimethamine, TRI—trimethoprim; SDZ—sulfadiazine. For details, see Table 1.

A summary of the ability of each TZD hybrid molecule (**1**–**12**) to inhibit the parasite’s growth by 50% (IC_50*Tg*_), the cytotoxic effect on the host cells (Hs27), expressed as the concentration that reduced the cells’ viability by 30% (as CC_30_), and the selectivity index (SI) calculated by CC_30_/IC_50*Tg*_ are presented in Table 1 and graphically illustrated in Figure 3, respectively. In these studies, pyrimethamine (PYR) and sulfadiazine (SDZ), typically used for the treatment of human toxoplasmosis [47], atovaquone (ATO), widely used for the treatment of severe toxoplasmosis, [48] and trimethoprim (TRI), commonly used in veterinary medicine [49], were used as positive controls. Dimethyl sulfoxide (0.1%) was used as the negative control-.

According to the results presented in Table 1, all the tested TZD-PCH hybrid molecules (**1**–**6**) with IC_50_ values in the range of 8.37–39.02 µg/mL showed anti-*Tg* activity that was 32- to 150-fold higher than SDZ (IC_50*Tg*_ = 1254.36 µg/mL). Among them, the most active molecules were molecule **2** with an IC_50*Tg*_ of 8.37 µg/mL and **1** with an IC_50*Tg*_ of 12.93 µg/mL. These compounds showed comparable or even better activity compared to TRI (IC_50*Tg*_ = 14.79 µg/mL). The introduction into **2** of an electron-donating methoxy (**3**) and ethoxy (**4**) group or an electron-withdrawing chloro (**5**) and bromo (**6**) substituent led, in all cases, to a decrease in anti-*Tg* activity. Within the series TZD-TSC (**7**–**12**), in turn, all the hybrid molecules were more effective than SDZ and TRI. In a similar manner to the TZD-PCH series (**1**–**6**), the TZD-TSC hybrid molecules with an unsubstituted phenyl ring, i.e., **10** (IC_50*Tg*_ = 5.32 µg/mL) and **7** (IC_50*Tg*_ = 5.94 µg/mL), were better *Tg* inhibitors, as compared to the substituted ones (**8**, **9**, **11** and **12**). Among the substituted hybrid molecules, the chloro-substituted phenyl ring, as in **8** (IC_50*Tg*_ = 8.74 µg/mL) and **12** (IC_50*Tg*_ = 10.17 µg/mL), was preferred over bromo **9** (IC_50*Tg*_ = 12.29 µg/mL) and methoxy **11** (IC_50*Tg*_ = 12.07 µg/mL) substitution. 

With respect to the cytotoxic effect against the host cells, all TZD hybrids (**1**–**12**) displayed negligible cytotoxicity towards the normal human foreskin fibroblast Hs27 cell line and all of them were not toxic to the host cells at anti-parasitic concentrations. Further calculation of the SI indexes showed that these compounds are promising for their anti-*Tg* activity, as indicated by a marked increase in their SI values, compared to SDZ, PYR, and TRI. Among them, molecule **7** with an SI > 148.28, **2** with an SI > 118.93, **8** with an SI > 110.67, and **12** with an SI > 94.59 are the most effective. Thus, it might be concluded that TZD hybrid compounds present comparably or even better anti-*Tg* potency compared to those recently reported hybrid compounds with thiazolidinone [50,51], TSC [52,53] or PCH [54] structural scaffolds.

### 2.3. In Vivo Toxicity Tests and Hemolytic Activity and Cytotoxic Effects on Host Cells

**Figure 4:**
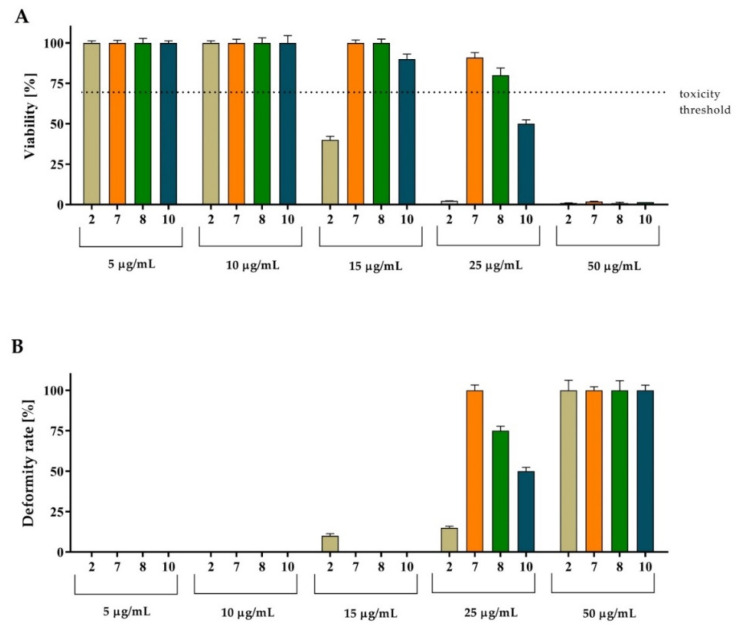
Overview of zebrafish embryo toxicity test after 96 h of exposure to TZD hybrid molecules **2**, **7**, **8**, and **10** (n = 30). (**A**) Toxicity of **2**, **7**, **8**, and **10** measured by the FET test at 96 hpf. (**B**) Impact of tested compounds on zebrafish development. Statistical significance was calculated using ANOVA analysis, followed by Dunnett’s post hoc test (vs negative control group). Concentrations above 50 μg/mL resulted in 100% mortality and deformity.

**Figure 5:**
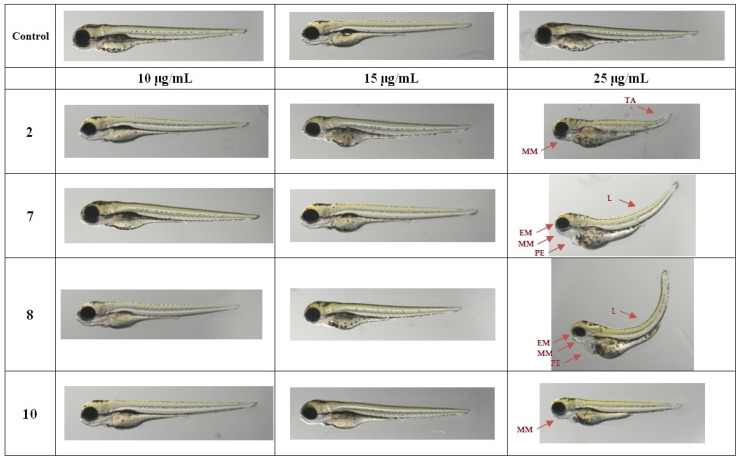
Effects of the investigated TZD hybrid molecules **2**, **7**, **8**, and **10** on zebrafish after 96 h of exposure. Representative morphological alterations in 96 hpf larvae are indicated by arrows. EM—eye malformations; HM—head malformations; MM—mandibular malformations; PE—pericardial edema; L—lordosis; TM—tail malformations.

To assess the cytotoxic effect of the most potent TZD hybrid molecules (**2**, **7**, **8** and **10**) on living organisms, the increasingly popular zebrafish model was used. Rapid development of Danio rerio embryos and the transparency of the larvae allow the assessment of drug-induced changes without the use of invasive techniques. The concentrations used for in vivo testing were selected based on the IC_50Tg_ values (5 µg/mL and 10 µg/mL). The results showed that in the therapeutic concentration range, the compounds **2**, **7**, **8** and **10** were not toxic to the zebrafish embryos and larvae within 96 h of observation. In the next step, higher concentrations were tested to determine the TZD hybrids’ effect on Danio rerio viability and development. Zebrafish embryos were exposed to the concentrations of 15, 25, 50, 75 and 100 µg/mL. The results showed that the compounds **7** and **8** had the smallest effect on the larvae viability (Figure 4). At the same time, the impact of these compounds on the occurrence of deformations was noticeable at a concentration of 25 µg/mL (i.e., 2.8–4.6-times higher than IC_50Tg_) (Figure 4 and Figure 5).

Finally, after the red blood cell treatment of molecules **2**, **7**, **8**, and **10**, no statistically significant increase in the level of free hemoglobin released to the medium was observed (Table 2), thereby indicating that the tested TZD hybrids at the concentration equal to IC_50*Tg*_ showed no specific hemolytic activity.

Each value is expressed as the mean ± SD (n = 3). The results were designated as statistically significant (ANOVA with post-hoc Dunnett’s test), when *p* < 0.05 (vs. untreated cells). Compounds **2**, **7**, **8** and **10** were tested at the concentrations that were equal to their IC_50*Tg*_ values.

### 2.4. Cytotoxic Effect against Human Glioblastoma T98G Cells

As a possible link between *Tg* infection and the risk of glioblastoma has recently been suggested [46], it was also of interest to characterize the cytotoxic potential of TZD hybrid molecules against human glioblastoma cells. For these studies, the most potent molecules (**2**, **7**, **8** and **10**) with experimentally confirmed ability to cross the blood–brain barrier (Table 3) were selected. According to the results presented in Table 4, compound **2** was found to be active against T98G glioblastoma cells with an IC_50_ of 19.36 ± 1.13 µg/mL. Although it demonstrates at least 5-fold higher activity than that of temozolomide, i.e., the anti-cancer agent routinely used in glioblastoma treatment, this concentration of **2** caused an impairment in the viability and functionality of living organisms, as shown in the zebrafish model. 

### 2.5. Clinical Significance of the Findings

This article describes pilot studies that explored the possible dual anti-infectious mode of action (anti-*Tg* and anti-*Mtb*) of TZD-based hybrid molecules. The most potent compounds were found to be more effective and more selective for *Tg* and *Mtb* pathogens than the reference drugs. Further studies for the selected candidates proved that they are non-cytotoxic, non-genotoxic, and non-hemolytic. Moreover, they could cross the BBB, which is a critical factor linked with ideal anti-*Tg* drug development. General conclusions from our studies suggest that within a series of TZD-based hybrid molecules, we are *able* to *find new* starting points for developing them into effective medicines to treat co-infections of *Tg* with *Mtb.*

## 3. Materials and Methods

### 3.1. Chemistry

TZD-PCH (**1**–**6**) and TZD-TSC (**7**–**12**) hybrid molecules were resynthesized by previously reported procedures [43,44,45]. All commercial reactants and solvents were purchased from either Alfa Aesar (Kandel, Germany) or Tokyo Chemical Industry Co. (Tokyo, Japan) with the highest purity and were used without further purification. Melting points (Gallenkamp MPD 350.BM 3.5 apparatus; Sanyo, Japan) and NMR spectra (Bruker Avance 300 MHz instrument with DMSO-*d_6_* as a solvent and TMS as an internal standard) were found in accordance with the literature data.

*General procedure for the synthesis of TZD-PCH (**1**–**6**) hybrid molecules*

To the mixture of 0.001 mol of corresponding formylphenyl (2,4-dioxothiazolidin-5-yl/ylidene)acetate and 0.001 mol of isonicotinic acid hydrazide, 8 mL of anhydrous ethanol was added. The mixture was heated under reflux until the substrates dissolved (15 min). After this, the mixture was cooled to room temperature and the precipitate was filtered off, dried and crystallized using butanol.

*General procedure for the synthesis of TZD-TSC (**7**–**12**) hybrid molecules*

To the mixture of 0.001 mol of corresponding formylphenyl (2,4-dioxothiazolidin-5-yl/ylidene)acetate and 0.001 mol of thiosemicarbazide, 5 mL of ethanol was added. The reaction mixture was heated under reflux for 15 min. After cooling, the precipitate was filtered off and washed with ethanol. After drying, the precipitate was recrystallized using the appropriate solvents (butanol or acetic acid).

### 3.2. Compound and Drug Preparation

TZD hybrid molecules (**1**–**12**) were dissolved in dimethyl sulfoxide (DMSO, Sigma-Aldrich, St. Louis, MO, USA) to reach the concentration of 150 mM. Sulfadiazine sodium salt (S6387, Sigma-Aldrich) was dissolved in DPBS without calcium chloride and magnesium chloride (Dulbecco’s Phosphate-Buffered Saline, D8537, Sigma) to reach 250 mM. Trimethoprim, pyrimethamine and atovaquone (92131, 46706 and A7986, Sigma-Aldrich) were dissolved in DMSO to reach 25 mM. The final concentration of DMSO in the compounds and drug dilutions was not higher than 1.00%. All hybrid molecules and drugs were freshly prepared before the experiment and sterile-filtered. 

### 3.3. Cell and Parasite Culture

The Hs27 cells (human fibroblast, ATCC^®^ CRL-1634™) were cultured in accordance with the ATCC product sheet in DMEM (Dulbecco’s Modified Eagle Medium, ATCC^®^ 30-2002™), supplemented with 10% fetal bovine serum (Fetal Bovine Serum, ATCC^®^ 30-2020™), 100 I.U./mL of penicillin and 100 μg/mL of streptomycin (Penicillin-Streptomycin Solution, ATCC^®^ 30-2300™). Cells were trypsinized (Trypsin-EDTA Solution, ATCC^®^ 30-2101™) twice a week and seeded at a density of 1 × 10^6^ per T25 cell culture flask (Corning^®^, Sigma-Aldrich) and incubated in 37 °C and 5% CO_2_ to achieve a confluent monolayer. The RH strain of *Toxoplasma gondii* (RH-GFP ATCC^®^ 50940™, highly virulent, haplogroup first; expression of green fluorescent protein) was maintained in the tachyzoite stage, according to the ATCC product sheet, in parasite culture medium, which contains DMEM medium with 3% HIFBS (heat-inactivated FBS; 1 h in 56 °C). Infected cells were incubated in 37 °C and 5% CO_2_.

### 3.4. In Vitro Anti-Tg Assay

The influence of the hybrid molecules **1**–**12** and drugs on *Tg* RH-GFP proliferation was analyzed as follows: 1 × 10^4^ per well of Hs27 cells were seeded on black, 96-well, tissue culture-treated plates with optical bottoms (Corning^®^) in DMEM cell culture medium. After 72 h of incubation, the medium was removed, and then 1 × 10^5^ per well of tachyzoite strains (RH-GFP) were added to the cell monolayers in the parasite culture medium. One hour later, the compounds and drug dilutions in the parasite culture medium were added to the Hs27 cells with *Tg*. The concentration of the compounds < CC_30_ were as follows: sulfadiazine, 0–10,000 μM; trimethoprim and pyrimethamine, 0–200 μM and atovaquone, 0–10 μM. After a subsequent 96 h of incubation, the plates were read with covered lids, and both excitation (475 nm) and emission (509 nm) values from the bottoms were obtained using the multi-mode microplate reader SpectraMax^®^i3 (Molecular Devices, San Jose, CA, USA). The results were expressed as the mean fluorescent intensity (MFI) and transformed to the percentage of viability compared to the untreated cells. Finally, the inhibitory concentrations for 50% inhibition of *Tg* proliferation (IC_50_) were calculated. All experiments were performed in triplicate.

### 3.5. Cytotoxicity Assay

Cytotoxicity assay was performed according to international standards (ISO 10993-5:2009(E)), using tetrazolium salt (MTT, Sigma-Aldrich) and mouse, as well as human, fibroblasts. For this assay, DMEM w/o phenol red (21063, Gibco, Thermo Fisher Scientific, Waltham, MA, USA) supplemented with 10% FBS, 100 I.U./mL of penicillin and 100 μg/mL of streptomycin was used. Briefly, 1 × 10^4^/well of Hs27 cells were placed into 96-well plates and incubated for 24 h in 37 °C and 5% CO_2_. Afterwards, an old culture medium was replaced by 100 μL of the compounds or drug dilutions in culture medium and the cells were treated for 24 h. The concentration ranges of the molecules were as follows: hybrid molecules **1**–**12**: 0–2500 μM, sulfadiazine: 0–25,000 μM, trimethoprim, pyrimethamine and atovaquone: 0–250 μM, and DMSO as the solvent: 0–20%. Then, 50 μL of 1 mg/mL of MTT solution in DMEM w/o phenol red was added to each well and incubated for 2 h (37 °C; 10% CO_2_). Following this, the cell culture medium was aspirated carefully, 150 μL of DMSO was added to each well and the plates were gently mixed. Then, 25 μL of 0.1 M glycine buffer (pH 10.5) (Sigma-Aldrich) was added. The optical density at 570 nm on the multi-mode microplate reader SpectraMax^®^i3 was read. The results were expressed as a percentage of viability compared to the untreated cells. All experiments were performed in triplicate.

### 3.6. Danio Rerio Culture and Fish Embryo Toxicity Test (FET)

*Danio rerio* (Experimental Medicine Centre, Medical University of Lublin, Poland) were maintained at 28 ± 0.5 °C, under a 14/10 h light/dark cycle with standard aquaculture conditions. After mating, the fertilized eggs were collected within 30 min. Embryos were reared in E3 embryo medium (pH 7.1–7.3; 17.4 µM NaCl, 0.21 µM KCl, 0.12 µM MgSO_4_ and 0.18 µM Ca(NO_3_)_2_) in an incubator (IN 110 Memmert GmbH, Germany) at 28 ± 0.5 °C. The eggs were examined to remove the unfertilized, coagulated and damaged samples. The FET test was performed based on the OECD Guidelines for the Testing Chemicals, Test No. 236. TZD hybrids were weighted, dissolved in DMSO as stock solutions, and diluted in E3 embryo medium to the indicated treatment concentrations. The stock of TZD hybrids and dilutions in E3 embryo medium were freshly prepared before testing. Embryos were incubated at each concentration (5, 10, 15, 25, 50, 75; 100 μg/mL) of TZD hybrids. The final DMSO concentration had no detectable effects on zebrafish development. The test was conducted in 24-well plates, with 5 embryos per well and 10 per group, in triplicate. The covered plates were kept at 28 ± 0.5 °C under the light/dark conditions (12 h/12 h). Embryonic viability, and the malformation rates of each treatment group were recorded at 24, 48, 72, and 96 hpf. All experiments were conducted in accordance with the National Institute of Health Guidelines for the Care and Use of Laboratory Animals and the European Community Council Directive for the Care and Use of Laboratory Animals (2010/63/EU). For the experiments on larvae up to 5 dpf, the agreement of the Local Ethical Commission is not required.

### 3.7. Hemolytic Activity Determination 

Human red blood cell (RBC) concentrate was obtained from the Regional Blood Donation and Transfusion Centre (Lublin, Poland). The RBC concentrate (5 mL) was washed three times with sterile PBS and centrifuged at 500× *g* for 3 min. The obtained pellet was resuspended using sterile PBS in order to obtain a 2% suspension of RBCs, which was subsequently mixed with 1 mL of different concentrations (i.e., 5, 10 and 35 mg/mL) of **2**, **7**, **8**, and **10**. The mixtures were incubated at 37 °C for 30 min and centrifuged at 1400× *g* for 10 min. The amount of free hemoglobin in the supernatants was measured spectrophotometrically at 405 nm. Negative and positive controls were performed by incubating RBCs with sterile PBS and 0.1% Triton-X, respectively. Each experiment was run in triplicate.

### 3.8. PAMPA-BBB Assay 

BBB permeability of **2**, **7**, **8**, and **10** was investigated using a parallel artificial membrane permeability assay (PAMPA) method. The PAMPA system, which consisted of a 96-well microfilter plate, was divided into the following two chambers: a donor at the bottom and an acceptor at the top, separated by a 120 µm thick microfilter disc coated with BBB lipid solution (Pion, Inc., Billerica, MA, USA). The solutions of **2**, **7**, **8**, and **10** were prepared in dimethylsulfoxide (DMSO) at 4 mg/mL concentration, and then diluted with Prisma buffer (pH = 7.4) to obtain the donor drug solution with the final nominal concentration of 20 µg/mL. Then, 180 µL of the donor solution was added to the donor wells. Subsequently, each filter membrane of the top plate was coated with 5 µL of BBB-1 lipid solution (Pion Inc., Billerica, MA, USA) and the acceptor well was filled with 200 µL of brain sink buffer (BSB). The acceptor plate and the donor plate were sandwiched together and incubated at 37 °C for 180 min. 

The permeability coefficient value (Pe) was calculated using the following equation:

$$P_{e}=\frac{-ln\;\left(1-\frac{C_{A}}{C_{equilibrium}}\right)}{S\times \left(\frac{1}{V_{D}}+\frac{1}{V_{A}}\right)\times t}$$

where *V_D_*—donor volume, *V_A_*—acceptor volume, *C_equilibrium_*—equilibrium concentration, $C_{equilibrium}=\frac{C_{D}\times V_{D}+C_{A}\times V_{A}}{V_{D}+V_{A}}$, *C_D_*—donor concentration, *C_A_*—acceptor concentration, *S*—membrane area and *t*—incubation time (in seconds).

### 3.9. Cytotoxicity against Human Glioblastoma T98G Cells

The T98G glioblastoma cells (ATTC, CRL-1690, Manassas, VA, USA) were cultured in Dulbecco’s Modified Eagle Medium (DMEM, high glucose) (Sigma Aldrich, St. Louis, MO, USA), supplemented with 10% heat-inactivated FBS, penicillin (100 U/mL), and streptomycin (100 U/mL) (Sigma Aldrich, St. Louis, MO, USA). Cells were maintained in a humidified atmosphere of 5% CO_2_ and 95% air at 37 °C. Stock solutions of the investigated compounds (**2**, **7**, **8** and **10**, and temozolomide) were prepared by dissolving solid substances in sterile DMSO. T98G glioblastoma cells were seeded into 96-well sterile plates at a density of 4 × 10^4^ cells/mL. After 24 h of incubation, the medium was removed from each well, and then the cells were incubated for the next 24 h with different concentrations of the investigated compounds (10, 25, 35, 50, and 100 mg/mL) in the medium that contained 2% FBS. Viability of the T98G cells was evaluated using MTT assay. In brief, after 24 h of incubation of the cells with varying concentrations, 15 μL of MTT solution (5 mg/mL) was added to each well. After 3 h of incubation, 100 mL (per well) of 10% SDS buffer solution was added. After overnight incubation, the absorbance was measured at 570 nm using a microplate reader (Epoch, BioTek Instruments, Winooski, VT, USA). Experiments were repeated three times, and the measurements in each experiment were run in quadruplicate. Viability of the investigated cells was expressed as % of the viability of the untreated cells. DMSO in the concentrations present in the dilutions of the stock solutions did not influence the viability of the tested cells. 

## 4. Conclusions

We report herein the anti-*Tg* characterization of TZD hybrid molecules that incorporated PCH (**1**–**6**) or TSC (**7**–**12**) structural fragments. By comparison of the biological data of TZD-PCH hybrids (**1**–**6**) with those of **7**–**12**, it emerges that TSC could be an important pharmacophoric requirement for anti-*Tg* activity. All the tested TZD-TSC hybrids (**7**–**12**) were more effective and more selective than SDZ and TRI. In addition, all of the hybrids were more selective than PYR. Further studies for the most potent TZD-TSC hybrids **7**, **8** and **10** and TZD-PCH hybrid **2** proved that the compounds at the concentrations equal to IC_50*Tg*_ are non-cytotoxic, non-genotoxic and non-hemolytic. Moreover, they could cross the BBB, which is a critical factor linked with ideal anti-*Tg* drug development. Finally, the anti-proliferative evaluation of **2**, **7**, **8**, and **10** disclosed the anti-cancer effect of **2** against human glioblastoma cells. Thus, the most potent TZD-based hybrid molecules could serve as a template for the development of novel therapeutics with dual anti-infectious (anti-*Tg* and anti-*Mtb*) modes of action and anti-glioma potency. 

## Figures and Tables

**Table 1:** Cytotoxicity (CC_30_), anti-*Tg* activity (IC_50*Tg*_), and selectivity index (SI) of TZD hybrid molecules (**1**–**12**) *^a^*.

TZD Hybrid Molecules		*CC_30_ ^b^* *in Hs27 Cells* (µg/mL)	*IC_50Tg_ ^c^ * *in Tg-Infected Hs27 Cells* (µg/mL)	SI ^*d*^
**1**	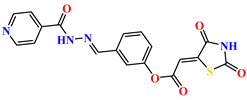	128.27	12.93	9.92
**2**	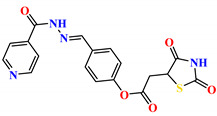	>995.98	8.37	>118.93
**3**	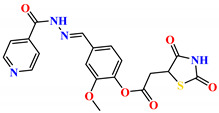	>1071.05	22.15	>48.35
**4**	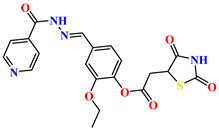	654.42	39.02	16.77
**5**	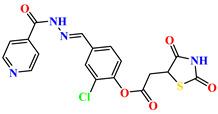	>1082.10	24.33	>44.48
**6**	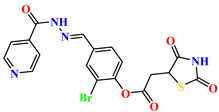	>1193.22	27.24	>43.81
**7**	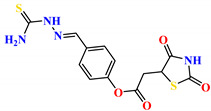	>880.97	5.94	>148.28
**8**	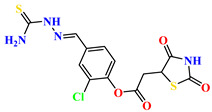	>967.09	8.74	>110.67
**9**	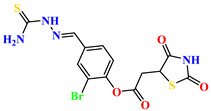	>1078.21	12.29	>87.75
**10**	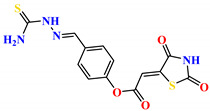	312.27	5.32	58.71
**11**	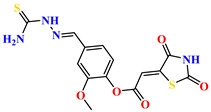	>951.00	12.07	>78.81
**12**	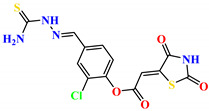	>962.05	10.17	>94.59
**ATO**	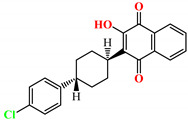	54.26	0.24	*227.55*
*PYR*	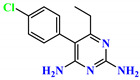	49.62	2.86	*17.38*
*TRI*	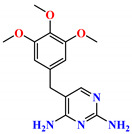	81.82	14.79	*5.53*
*SDZ*	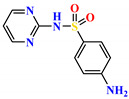	2230.61	1254.36	*1.78*

*^a^* Each value is expressed as the mean ± SD (n = 3). *^b^* CC_30_—the highest dilution of the samples to reduce the viability of the Hs27 cells by 30%. *^c^ IC_50Tg_*—the concentration required to reduce *Tg*-infected Hs27 cell growth by 50%. *^d^* SI—selectivity index calculated by CC_30_/IC_50_. Reference drugs: ATO—atovaquone, PYR—pyrimethamine, TRI—trimethoprim; SDZ—sulfadiazine.

**Table 2:** Effect of TZD hybrid molecules **2**, **7**, **8** and **10** on red blood cell hemolysis.

TZD Hybrid Molecules	Hemolysis (%)
**2**	0.68 ± 0.09
**7**	0.73 ± 0.1
**8**	0.69 ± 0.04
**10**	0.66 ± 0.02
Untreated cells	0.59 ± 0.06
Triton-X (0.1%)	99.85 ± 1.65 ****

****—*p* < 0.0001.

**Table 3:** PAMPA-BBB permeabilities of TZD hybrid molecules **2**, **7**, **8**, and **10**.

TZD Hybrid Molecules	PAMPA-BBB P_e_ (×10^−6^ cm·s^−1^) *^a^*
**2**	12.45 ± 1.02
**7**	23.05 ± 0.71
**8**	26.11 ± 1.30
**10**	24.37 ± 0.93

*^a^* P_e_—permeability values. Data are the mean of three independent experiments ± SD. High BBB permeation (CNS+) is expected for compounds with P_e_ > 5.19, whereas low BBB permeation (CNS−) is expected for compounds with Pe < 2.07.

**Table 4:** Cytotoxic effect of TZD hybrids **2**, **7**, **8**, and **10** against human glioblastoma T98G cells.

IC_50_ (µg/mL) ± SD				
**2**	**7**	**8**	**10**	**Temozolomide**
19.36 ± 1.13	>100	>100	94.57 ± 6.16	>100

## Data Availability

The data presented in this study are available on request from the corresponding author (N.T.)
